# Energy-related nanomaterials

**DOI:** 10.3762/bjnano.4.76

**Published:** 2013-10-24

**Authors:** Paul Ziemann, Alexei R Khokhlov

**Affiliations:** 1 Institute of Solid State Physics, Ulm University, D-89081 Ulm, Germany; 2 Faculty of Physics, M.V. Lomonosov Moscow State University, Moscow, Russia

**Keywords:** energy related, nanomaterials

The triple “generation, conversion and storage of energy” are the fundamental building blocks toward realizing the general aim of an energy supply on demand as unrestricted as possible. Meanwhile, however, it has become clear that this general aim leads to conflicting feedback loops on the ecological environment. Growing awareness of such deteriorating feedbacks has triggered worldwide activities to search for technical approaches in order to at least reduce the negative environmental consequences of energy consumption. At this point, materials science plays a central role. In most cases, rather than looking for completely novel solutions related to the energy triple “generation, conversion and storage” materials science contributes to stepwise improvements of functional efficiencies, which are based on optimizing energy-related materials.

A well-known topic related to the transport of people and goods – an important subsector of human energy consumption – is the effort aimed at the reduction of the weight of cars, trucks, planes and ships in order to save fuel. Improved materials such as novel compounds or composites should not only result in a reduction of weight but should also have mechanical properties that are at least comparable to those of the materials they are exchanged for. Mechanical properties strongly depend on the internal structure of a material such as grain size, grain orientation, defect densities, local chemical composition, and layer sequence and thickness in layered systems. At this point, the micro- and nanometer scales enter both experimental and theoretical simulation routes towards materials optimization. Another materials property worth of being optimized is friction, which, when trying to walk or drive on icy streets is highly welcome, but in many cases is a source of dissipated energy. The friction of an object moving on or through a supporting or surrounding medium, respectively, appears to be closely related to surface or interface roughness, so that, again, the micro- and nanoscales are of major importance. For instance, a reduction of the friction of cargo vessels by only a few percent leads to considerable energy savings and a significant decrease of the worldwide CO_2_ emission [1]. It is noteworthy that the nanopatterning of surfaces and interfaces to reduce friction by tailoring their wettability and anti-fouling behavior is often guided by mimicking nature [2–3]. Contributions of advanced materials science to energy savings are numerous even when restricting one’s view just on personal surroundings: Intelligent housing with controlled energy delivery, distribution and consumption, including smart heat production and insulation as well as smart windows complemented by exploiting residual heat.

Besides energy savings to extend finite resources, negative ecological feedbacks demand alternatives to oil-derived fuels. As we have already outlined previously [4], this demand is particularly important in the context of mobility with its significant subsector of short and medium range transport of people and products. Despite the increasing efforts to implement public transit and public transportation systems, cars and trucks with conventional combustion engines are still the predominant means of transport causing a considerable contribution to the global CO_2_ emission. Electrically powered vehicles can at least contribute to attenuate this emission problem.

Electrically powered vehicles strongly rely on fuel cell (FC) or, most importantly, lithium-ion battery (LIB) technology, which is well-known and is already used on a large scale. However, the efficiency and average lifetime of these devices is suboptimal, and new materials are needed to fulfill these requirements. The development of such materials is a challenging task for experimental and theoretical materials science, which can only be met by joint interdisciplinary efforts between physicists, chemists and engineers. In addition, energy storage poses an even greater challenge, requiring contributions from the fields of electrochemistry, catalysis and simulations on all length scales.

Therefore, the cooperation of research facilities with a long-established experience in materials-oriented chemistry including catalysis and electrochemistry is a logical step, enabling the participating scientists to join their complementary knowledge and experience. The gains of such a multidisciplinary team play was the driving force for the initiation of joint projects between Ulm University, Moscow State (Lomonosov) University, and the Russian Academy of Sciences, financially supported by a recently completed BMBF-i program that supports the installation of a joint research infrastructure between German and Russian partners [4].

The contributions to the Thematic Series “Energy-related nanomaterials” exemplify the scientific outcome of this cooperation and are complemented by related work outside of this network. Emphasis is put on a balanced presentation of experimental and theoretical work as well as physics- and chemistry-oriented views and approaches. The contributions cover a plethora of research fields from materials-related problems that concern fuel cells, Li-based batteries, and organic solar cells, to energy-related applications of nanographite and silicon nanotubes as well as the optimization of thermoelectric materials and electrochemistry-based microscopy.

We would like to thank all colleagues for their valuable contributions and are convinced that this Thematic Series will find many readers. The engaged editorial support by the Production Team of the Beilstein-Institut is greatly acknowledged.

Paul Ziemann and Alexei R. Khokhlov

Ulm, Moscow, September 2013

## References

[1] Corbett J J, Koehler H W (2003). J Geophys Res: Atmos.

[2] Barthlott, W.; Koch, K., Eds. Biomimetic materials, Beilstein J. Nanotechnol.

[3] Barthlott W, Koch K (2011). Beilstein J Nanotechnol.

[4] ITP Infoservice 01/2012 - 5. Schwerpunktausgabe: Russland – Modernisierung durch Innovation und Forschung.

